# Antibiotic prescription rationality and associated in-patient treatment outcomes in children under-five with severe pneumonia at Bwizibwera health center IV, Mbarara District, South-Western Uganda

**DOI:** 10.1186/s41479-022-00095-0

**Published:** 2022-04-25

**Authors:** Christine Joy Abeja, Vallence Niyonzima, John Paul Byagamy, Celestino Obua

**Affiliations:** 1 Department of Nursing and Midwifery, Lira University, Lira, Uganda; 2 Department of Nursing, Mbarara University of Science and Technology, Mbarara, Uganda; 3 Department of Public Health, Lira University, Lira, Uganda; 4 Office of the Vice Chancellor, Mbarara University of Science and Technology, Mbarara, Uganda

**Keywords:** Antibiotic, Children, Rationality, Pneumonia, Prescription, Treatment outcomes, Bwizibwera, Health center IV

## Abstract

**Background:**

Pneumonia is a major cause of morbidity and mortality in children under five. Antibiotic treatment must be started immediately in children with pneumonia. The irrational use of antibiotics may increase morbidity and mortality in children with pneumonia. Pneumonia accounted for approximately 16% of the 5.6 million under-five deaths word wide in 2016. In Uganda, it kills approximately 2,400 children per year. Early diagnosis and appropriate case management with rational use of antibiotics remain the most effective intervention to reduce pneumonia-related mortality. This study aimed at determining antibiotic prescription rationality and associated in-patient treatment outcomes in children aged 2–59 months with severe community-acquired pneumonia at Bwizibwera Health Centre IV from 1^st^ May 2018 to 30^th^ April 2019.

**Methods:**

We conducted a retrospective cohort study design; data were collected from in-patient records of all children aged 2–59 months with severe community-acquired pneumonia who met the eligibility criteria for a period of one year. Data abstraction template was used for data collection. Health care records of children aged 2–59 months who had other co-morbidities and were on medication that could influence or impact on in-patient treatment outcomes from 1st May 2018 to 30th April 2019 were excluded. Data was entered and analyzed using Epi-info v 7.2 and STATA v 13.0 respectively, Descriptive statistics were reported and Chi-square test was used to compare the proportions.

**Results:**

Of the total records of children retrieved and screened (*N* = 847), 229 prescription records of children fulfilled inclusion criteria, 57 (24.9%) had rational prescriptions with good outcomes and 172 (75.1%) had irrational prescriptions with 10 (4.4%) having unfavorable outcomes. The majority (73.7%) of those who received rational prescription were on treatment with a combination of benzyl penicillin plus gentamycin while (26.3%) were on ampicillin plus gentamycin. The majority (32.4%) of patients with good treatment outcomes were aged 6 – 11 months. This age category also doubled as the group that experienced the highest percentage (40.0%) of unfavorable outcomes. There were no statistically significant associations between patient characteristics and treatment outcomes.

**Conclusion:**

In conclusion, the majority of children had irrational antibiotic prescriptions and 40 percent of children aged 6–11 Months had unfavorable treatment outcomes with 20 percent death. This study also found out that majority of antibiotic prescription among children under five was irrational and it’s against Uganda clinical guideline for treatment of severe pneumonia among children under five.

## Background

**Fig. 1:**
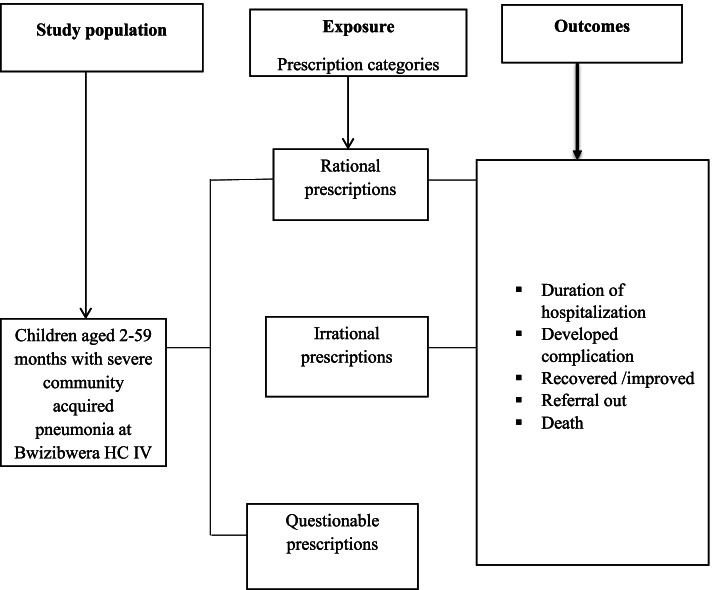
Flow chart showing study design

**Fig. 2:**
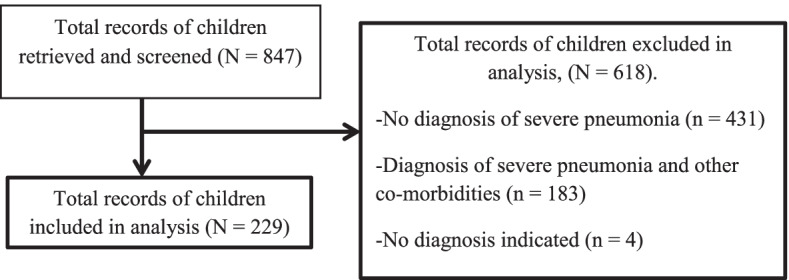
Study profile

**Fig. 3:**
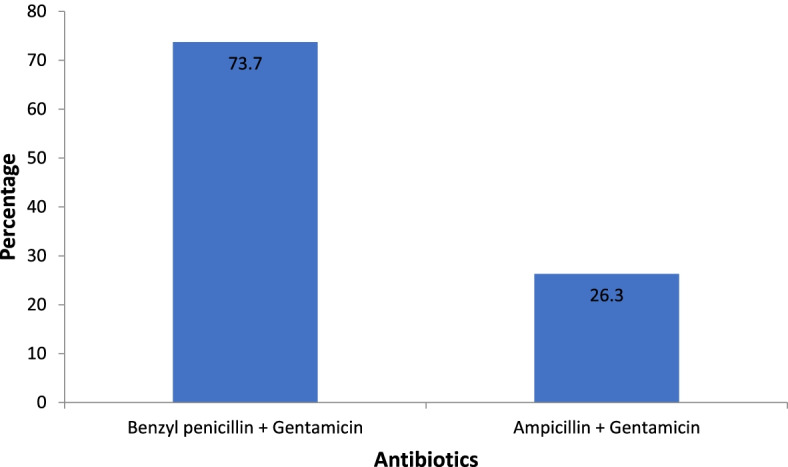
Graph showing the recommended first line regimens used for treatment of severe pneumonia

**Fig. 4[4]:**
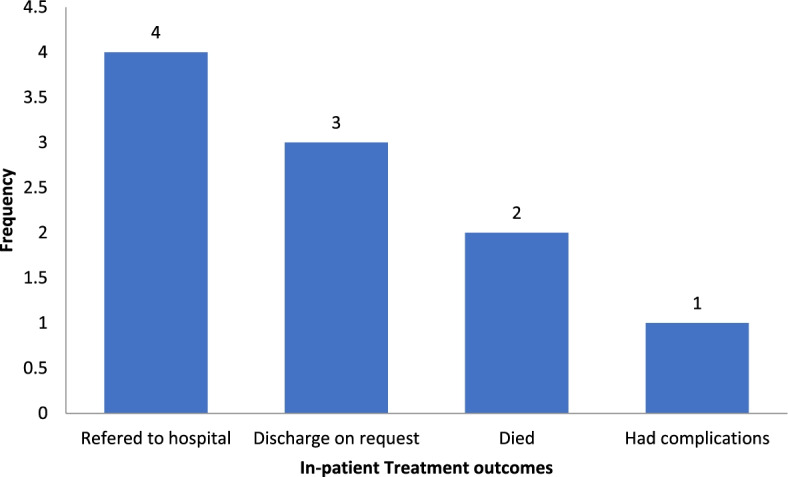
Irrational prescriptions of antibiotics with unfavorable treatment outcomes

Pneumonia is a form of acute respiratory infection that affects the lungs. When an individual has pneumonia, the alveoli are filled with pus and or fluid, which make breathing painful and limits oxygen intake [1, 2]. In 2016, pneumonia accounted for approximately 16% of the 5.6 million under-five deaths, killing around 880,000 children, most of these children were less than 2 years [2]. In 2015, pneumonia killed 920,136 children under the age of five, accounting for 16% of all deaths in this age group and is most prevalent in South Asia and sub-Saharan Africa [3]. In Uganda, it kills approximately 2,400 children per year [4] (Figs. 1, 2, 3, 4).

Early diagnosis and appropriate case management with rational use of antibiotics remain the most effective intervention to reduce pneumonia-related mortality. Unfortunately, critical inequities in the access to antibiotics and health services exist in most developing countries, often leading to irrational medicines use, irrational antibiotic use involves incorrect indications, at incorrect doses, or for inappropriate durations [5]. Irrational use of antibiotics can take different forms; inappropriate prescriptions, over and under-prescribing, Polypharmacy, unreasonable use of expensive medicines and inappropriate use of antibiotics [6]. However other studies have also reported irrational use of antibiotics in children under five years with severe pneumonia [7–10] Antibiotics are among the most prescribed medicines among in-patient children, with almost half the prescriptions being irrational [3]. The irrational use of antibiotics may increase morbidity and mortality in children with pneumonia [4]. Rational use of antibiotics is important element in attaining quality of health and medical care [5]. The Uganda Clinical Guidelines (UCG) recommends that, the first line antibiotic regimen for treating severe pneumonia in children under 5 years is; Ampicillin 50 mg/kg body weight Intravenous (IV) 6 hourly or Benzyl penicillin 50,000 IU/kg body weight Intramuscular (IM) or IV plus Gentamicin 7.5 mg/kg body weight IM or IV once daily. This regimen is given for at least 5 days to 10 days [11]. If there is no improvement after 48 h, the second line must be administered, that is, Ceftriaxone 80 mg/kg IM or IV once daily. Once the patient improves, he/she is switched to oral Amoxicillin 40 mg/kg body weight 12 hourly for 5 days in order to complete a total of at least 5 days [11]. Inappropriate antibiotics use has resulted in increase in development of drug resistance pathogens with high implication in terms of morbidity and mortality [6]. Several studies revealed decreased prescriptions of antibiotics among children, whilst the use of broad-spectrum antibiotics increased [7, 8]. Antibiotics are the most commonly prescribed medicines in hospitals, However, excessive and inappropriate use of antibiotics leads to increased drug resistance [9, 10, 12–15]. Several studies around the globe reported unfavorable treatment outcomes in children under five with irrational prescriptions of antibiotics [15–19]. There is limited data on the rationality of antibiotic prescriptions and its associated in-patient treatment outcomes in Mbarara District. The aim of this study was to assess antibiotic prescription rationale and its associated in-patient treatment outcomes for children under five years with severe community-acquired pneumonia at Bwizibwera Health Center IV, Mbarara District, in South-Western Uganda.

## Methods

### Study setting

The study was conducted at Bwizibwera Health Centre IV, Mbarara District in South-western Uganda. Bwizibwera H/C IV is a primary care facility owned by the government of Uganda. It is located about 25 kms from Mbarara town on Ibanda road. Bwizibwera H/C IV has two medical officers, two clinical officers, a team of midwives and nurses who offer diagnostic and treatment services for common illnesses. The same team of health workers and support staff also provide antenatal care, acute medical, emergency obstetric care and surgical services. The Health Centre provides medical services free of charge to patients. The medicines in this Health facility are supplied by the National Medical Stores under the supervision of MOH. Antibiotics are among the most frequently prescribed medicine in this Health facility.

## Design of the study

A retrospective cohort study was carried out for a period of one year (May 2018 to April 2019), this design was chosen because it minimizes the rate of change in behavior for prescribers that is likely to happen in a prospective cohort.

The prescription rationality was evaluated in reference to the Uganda Clinical Guidelines [11] and a modified criteria set by Badar et al.  [10].

A prescription was rational if the antibiotic prescribed is appropriate for the indication, in the right dose, frequency, duration and route of administration as guided by the UCG [11].

A prescription was irrational, if it was not appropriate for any one of the following; the indication, dose, frequency, duration and route of administration as guided by the UCG.

## Study population

These were records from in-patient department treatment register of children aged 2–59 months with severe community acquired pneumonia who were admitted from 1^st^ May 2018 to 30^th^ April 2019.

## Data collection procedure

The data extraction form was developed in reference to the UCG on the management of severe pneumonia and the criteria for evaluating the prescriptions was used to record all information about each child from the selected records. The data extraction tool had information on the child’s social demographic, clinical presentation on admission, date of admission, and subsequent days of follow-up, duration of hospitalization, complications, referrals out of the facility/date of discharged, prescribed treatment regimens on the daily basis that was evaluated for; rationality, de-escalation, change from intravenous to oral regimens, and finally deaths. Data was retrieved by the principal investigator and trained research assistants.

## Data analysis

Data was entered and analyzed using Epi-info v 7.2 and STATA v 13.0 respectively. Descriptive statistics were reported and Chi-square test was used to compare the proportion.

### Ethical considerations

Ethical clearance was obtained from Faculty of Medicine Research Committee (FRC) and MUST-Research Ethics Committee (REC). Permission was sought from the District Health Officer and the in-charge Bwizibwera H/C IV to access health care records of children aged 2–59 months prior to data collection. Confidentiality was ensured by coding the data abstraction templates instead of using names. The data collected were kept under key and lock and accessed by the research team only. Soft data was kept in a computer with a password known only by the research team

## Results

A total of 847 records of children were retrieved and screened, 229 records were included for data collection and analysis. A total of 618 records of children were excluded as follows; 431 records had no diagnosis of severe pneumonia, 183 records had a diagnosis of severe pneumonia with other comorbidities and 4 records had no diagnosis indicated.

## Socio-Demographic Characteristics

From Table 1 above; the mean age of children was 15.6 months, with the largest group 75 (32.8%) between the age of 6–11 months. The majority of children 119 (52.0%) were female. Female care takers dominated being next of kin 212 (92.6%), and only 6 (2.6%) were referred from lower health facilities.

**Table 1:** Socio-demographic characteristics of the study population

Characteristic *N* = 229	Frequency, *n* (%)
**Mean age in months** (SD)	15.6 (11.9)
**Age categories in months** 2–5 6–11 12–23 24–59	35 (15.3) 75 (32.8) 58 (25.3) 61 (26.6)
**Gender** Female Male	119 (52.0) 110 (48.0)
**Sex of next of kin** Female Male	212 (92.6) 17 (7.4)
**Referred from lower health facility** No Yes	223 (97.4) 6 (2.6)

**Table 2:** Antibiotic prescription rationality

Rational prescription	Frequency n (%)
No Yes **Total**	172 (75.1) 57 (24.9) **229 (100)**

## Proportion of children 2–59 months that received rational antibiotic prescriptions

In this analysis, a child was considered to have rational antibiotic prescription if he or she received right regimen for right: indication, route of administration, dose, dose frequency, duration of treatment [11] Table 2.

Out of the 229 children whose records were retrieved and analyzed, 57 (24.9%) had rational prescription and were treated with the recommended first line antibiotic combinations for treatment of severe pneumonia i.e. Ampicillin plus Gentamicin or Benzyl penicillin plus Gentamicin.

Of the 57 rational prescriptions, 42 (73.7%) were of Benzyl penicillin plus Gentamycin while the other 15 (26.3%) were of Ampicillin plus Gentamycin Table 3.

**Table 3:** Components of rational antibiotic prescription

Variable	Frequency n (%)
**Right regimen**	
No Yes	172 (75.1) 57 (24.9)
**Duration (continued for at least 5 days)** No Yes	40 (70.2) 17 (29.8)
**Frequency of administration** No Yes	3 (5.3) 54 (94.7)

The majority 172 (75.1%) did not have a rational prescription for regimen. while 57 (24.9%) had it. Of those with rational prescription, only 17 (29.8%) had a rational duration prescribed as per records. Further still, most 54 (94.7%) had a rational prescribed frequency of administration Table 4.

**Table 4:** Rationality of antibiotic prescription by age and gender

Variable	Irrational prescription, n (%)	Rational prescription, n (%)	P-value
**Age categories (months)** 2–5 6–11 12–23 24–59	31 (18.0) 44 (25.6) 47 (27.3) 50 (29.1)	4 (7.0) 31 (54.4) 11 (19.3) 11 (19.3)	0.347
**Gender** Female Male	92 (53.5) 80 (46.5)	27 (47.4) 30 (52.6)	0.671

## Stratification of rational antibiotic prescription by age and gender

Rational antibiotic prescription was stratified by age. Analysis revealed that of the 57 with rational prescriptions, the majority (54.4%) were between the ages of 6–11 months. Of those with irrational prescriptions, the majority (29.1%) were aged between 24–59 months. There was no statistical significant association between rational and irrational antibiotic prescription with age and gender with the *P*-value of 0.347 and 0.671 respectively.

## In-Patient treatment outcomes

In this analysis, a child was considered to have unfavorable outcome if he or she had any of the following outcomes: developed complication, referred to hospital, died, or were discharge on request. A child was considered to have a good outcome if he or she had improved and discharged home (See Table 5).

**Table 5:** In-patient treatment outcomes

Condition at discharge	Irrational n (%)	Rational n (%)
Good outcomes Unfavorable outcomes	162 (94.2) 10 (5.8)	57 (100) 0 (0)

Of the 229 analyzed records of children on rationality of antibiotic prescription and treatment outcomes, 10 (5.8%) had unfavorable treatment outcomes with irrational prescriptions. The commonest unfavorable outcomes were referral to hospital and discharge on request (See Fig. 4) Table 6.

**Table 6:** Socio-demographic characteristics and treatment outcomes

Demographic Characteristics	Good outcome *n* (%)	Unfavorable outcome n (%)	P value
**Age categories (months)** 2–5 6–11 12–23 24–59	32 (14.6) 71 (32.4) 58 (26.5) 58 (26.5)	3 (30.0) 4 (40.0) 0 (0.0) 3 (30.0)	0.227
**Gender** Female Male	114 (52.0) 105 (48.0)	5 (50.0) 5 (50.0)	0.899

## Association between irrational antibiotic prescriptions with in-patient treatment

In relation to age, majority of children aged 6–11 months 71(32.4%) had good outcomes. Similarly, they had highest number of those with unfavorable outcome. Females had slightly better outcomes 114 (52.0%), compared to males. The majority (97.7%) of children with good outcomes were none referred from lower health facilities.

## Discussion

This study aimed at determining the rationality of antibiotic prescriptions and associated in-patient treatment outcomes in children aged 2–59 months with severe pneumonia at Bwizibwera Health Center IV, Mbarara District in south-western Uganda from 1st May 2018 to 30th April 2019.

In this study, 75.1% of antibiotic prescriptions were irrational among children under five in Bwizibwera HC IV, Mbarara District. Another study conducted in Western Uganda revealed 61.9% of antibiotic prescriptions [5]. These high percentages could be attributed to availability of antibiotics at the time of prescription. Our study was conducted in Government health facility with frequent stock out of essential medicines among others antibiotics, while in the other study of Akunne [5] was done in a private hospital with availability of antibiotics of choice.

Earlier studies found that despite the availability of treatment guidelines, Uganda’s health care system is still challenged with high rates of irrational antibiotic use [17–19]. This could be due to poor implementation of government policies and guidelines which at the end affects rationality of antibiotics prescription.

In the studies conducted in Turkey and Mongolia slightly smaller percentages of irrational antibiotic prescriptions were reported (56.5%) and (56.6%) respectively [7, 8]. The study population in Turkey and Mongolia were higher compared to our current study population in Uganda. This could preliminarily explain the observed differences.

In other countries, irrational prescriptions of antibiotics were observed among children [3, 6, 9] of 33.4%, 35.1% and 46% respectively. These differences could be accorded to difference in geographical location, treatment guidelines, competence of prescribing staff and availability of antibiotics.

Our study revealed that 24.9% of antibiotic prescriptions were rational. This was based on the right regimen, right duration and frequency of drug administration. The percentage of rational antibiotic prescriptions in the current study is lower than that reported earlier in Tanzania of 44% [12].The difference in the reported percentage of rational prescriptions by the study in Tanzania and our study could have risen due to the fact that the Tanzanian study was a multi-center study and involved patients with several disease conditions other than just pneumonia.

A study conducted in public health care facilities in Uganda reported that rational prescription was 12.4% [14] and this is lower than the 24.9% revealed by our study. The different in percentages could be due to antibiotic prescription in one condition and in one health facility while Trap et al., [11] looked at all levels of health care facilities in Uganda and the general performances in the country.

In Turkey, the rate of rational antibiotic use was reported to be 11.3% [7]. While in our current study rational antibiotic prescription was 24.9%. This could be attributed to difference in treatment guidelines between Uganda and Turkey and also difference in study design.

The outcomes of antibiotic treatment were categorized into good and unfavorable outcomes in the current study. A child was considered to have a good treatment outcome if he or she improved and was discharged within 7 days.

Unfavorable treatment outcomes were considered when there was development of complications, referral to the hospitals, self-discharged and deaths.

This study reported 2 deaths (20%) out of 10 unfavorable outcomes which is comparable with a study in Indonesia of 7 (15.2%). This borderline similarity could be due to poor choice of the antibiotics, wrong dosage, dose and route of administration and other empirical error.

## Conclusion

In conclusion, the majority of children had irrational antibiotic prescriptions and 40 percent of children aged 6–11 Months had unfavorable treatment outcomes with 20 percent death. This study also found out that majority of antibiotic prescription among children under five was irrational and it’s against Uganda clinical guideline for treatment of severe pneumonia among children under five.

## Data Availability

All data generated and analyzed during this study are included in this article.

## Abbreviations

ARI: Acute Respiratory infection
CAP: Community-Acquired Pneumonia
DHO: District Health Officer
EIN: Emerging Infections Network
H/C: Health Centre
IM: Intramuscular
IV: Intravenous
MMS: Medicine Management Supervisors
MOH: Ministry of Health
SPARS: Supervision, Performance Assessment, and Recognition Strategy
UCG: Uganda Clinical Guidelines
UNICEF: United Nations International Children’s Emergency Fund
WHO: World Health Organization
FRC: Faculty Research Committee
REC: Research Ethics Committee
